# Photo-Excited Carrier Dynamics in Ammonothermal Mn-Compensated GaN Semiconductor

**DOI:** 10.3390/ma17235995

**Published:** 2024-12-07

**Authors:** Patrik Ščajev, Paweł Prystawko, Robert Kucharski, Irmantas Kašalynas

**Affiliations:** 1 Institute of Photonics and Nanotechnology, Faculty of Physics, Vilnius University, Saulėtekio Ave. 3, 10257 Vilnius, Lithuania; 2 Laboratory of Semiconductor Characterization, Institute of High Pressure Physics PAS (UNIPRESS), ul. Sokołowska 29/37, 01-142 Warsaw, Poland; pprysta@unipress.waw.pl (P.P.); robert.kucharski@unipress.waw.pl (R.K.); 3 Terahertz Photonics Laboratory, Center for Physical Sciences and Technology, Saulėtekio Ave. 3, 10257 Vilnius, Lithuania; 4 Institute of Applied Electrodynamics and Telecommunications, Vilnius University, Saulėtekio Ave. 3, 10257 Vilnius, Lithuania

**Keywords:** gallium compounds, wide-bandgap semiconductors, ammonothermal GaN:Mn, photoluminescence decays, pump–probe decays, time-resolved photo-carrier transport

## Abstract

We investigated the carrier dynamics of ammonothermal Mn-compensated gallium nitride (GaN:Mn) semiconductors by using sub-bandgap and above-bandgap photo-excitation in a photoluminescence analysis and pump–probe measurements. The contactless probing methods elucidated their versatility for the complex analysis of defects in GaN:Mn crystals. The impurities of Mn were found to show photoconductivity and absorption bands starting at the 700 nm wavelength threshold and a broad peak located at 800 nm. Here, we determined the impact of Mn-induced states and Mg acceptors on the relaxation rates of charge carriers in GaN:Mn based on a photoluminescence analysis and pump–probe measurements. The electrons in the conduction band tails were found to be responsible for both the photoconductivity and yellow luminescence decays. The slower red luminescence and pump–probe decays were dominated by Mg acceptors. After photo-excitation, the electrons and holes were quickly thermalized to the conduction band tails and Mg acceptors, respectively. The yellow photoluminescence decays exhibited a 1 ns decay time at low laser excitations, whereas, at the highest ones, it increased up to 7 ns due to the saturation of the nonradiative defects, resembling the photoconductivity lifetime dependence. The fast photo-carrier decay time observed in ammonothermal GaN:Mn is of critical importance in high-frequency and high-voltage device applications.

## 1. Introduction

Gallium nitride (GaN) is a wide-bandgap semiconductor suitable for electronic high-frequency and high-power devices, possessing high values of electron velocity, high breakdown field strength, and good thermal conductivity [1]. Thus far, most GaN-based devices are built on foreign substrates such as silicon, silicon carbide, and sapphire, leading to large lattice and thermal mismatches and, therefore, high defect densities on the interfaces [2]. These mismatches could be fully avoided using native GaN substrates. For many years, native substrates were not available in large quantities on the market due to the challenging GaN crystal growth process. The compound melts at extremely high temperatures (>2200 °C), and the nitrogen pressure necessary for the congruent melting of GaN is expected to be higher than 6 GPa [3,4]. Thus, it is currently impossible to crystallize GaN from a melted state. This compound should be grown using other techniques that require lower pressures and temperatures. Crystallization from the gas phase, a solution, or any combination thereof must be included. For more than the last three decades, the growth of bulk GaN has remained challenging for the nitride community.

Significant progress has been made with the ammonothermal method; by using both acidic and basic approaches, GaN crystals can be grown for large-scale production and scalability [5], obtaining high-structural-quality 4-inch-diameter crystals [6]. The basic ammonothermal technique allows for the crystallization of semi-insulating (SI) and highly conductive n-type crystals [6]. Compensation was obtained by using C or Mn acceptors. Mn provides the best GaN properties and can be used for the further development of semi-insulating GaN devices. Recently, ammonothermal Mn-doped GaN (Am-GaN) has revealed extremely large GΩ resistances at the kilovolt level [7] and was demonstrated to be suitable for the development of ultrafast sub-bandgap high-voltage GaN switches [8]. Moreover, Mn doping has been applied for the production of SI GaN solar cells with an Mn intermediate band [9] and photo-electrodes for the photo-electrolysis of water under visible light [10]. Thus, research and development into sub-bandgap vertical GaN devices may evolve with an improvement in the growth processes and a reduction in unintentional impurity density.

For high-power and high-voltage operations, vertical device architectures are being proposed and investigated [11], for which SI GaN has a high potential for practical applications. Contrary to other semiconductors, GaN is a relatively young material used for the development of efficient electro-optical modulators [12] and high-frequency sources [13,14]; thus, its degradation and charge-trapping processes are not well understood and described, showing the need for improvements in device reliability. Therefore, in this work, we investigate the carrier dynamics of Am-GaN:Mn using time-resolved photoluminescence, absorption methods, and a comparison with the photoconductivity (PC) technique [8] to reveal the carrier transition processes through the Mn states and propose further development of this material. We enrich the understanding of the temporal photoconductivity processes observed in the Mn states [8] and of the femtosecond pump–probe in the Mn band tails [15] by conducting contactless time-resolved photoluminescence (PL) analysis and nanosecond pump–probe (PP) investigations, providing more details about the impact of Mn on photo-carrier relaxation.

## 2. Materials and Methods

A two-side polished, semi-insulating Am-GaN sample of 380 μm thickness was used for the investigation [8]. The sample was grown ammonothermally in the temperature range of 500–600 °C and under a pressure of 0.3–0.4 GPa in the -c direction. The growth was conducted in a Ni-based alloy autoclave from GaN seeds with low dislocation density, <5 × 10^4^ cm^−2^ [16]. The resistivity of the Am-GaN sample exceeded 10^11^ Ω × cm. The sample contained the following impurities due to residual doping in a high-pressure growth process: Mn, 1 × 10^19^ cm^−3^; H, 2 × 10^19^ cm^−3^; O, 6 × 10^18^ cm^−3^; C, 4 × 10^17^ cm^−3^; Si, 2 × 10^17^ cm^−3^; Mg, 2 × 10^17^ cm^−3^ [16]. A 10 μm thick Si-doped n-type GaN epilayer with 2 mS × sq. sheet conductance and 10^16^ cm^−3^ electron density was deposited on the Ga-face (0001) of the sample via the MOVPE in the Close Coupled Showerhead^®^ (CCS) Aixtron 3 × 2-inch flip-top (FT) reactor (Aachen, Germany) [17] and served as a transparent conductive top contact layer for laser excitation [8]. For the photoconductivity measurements, Ag contacts were deposited on both sides of the sample, retaining an opening in the center of the layer side [8].

The photoluminescence (PL) spectra and decays were recorded using a Hamamatsu streak camera (C10627, Hamamatsu, Japan) and an Acton monochromator (SP2300, Princeton Instruments, Acton, MA, USA) at a pulsed laser excitation provided by an optical parametric generator pumped with a Yb:KGW laser (PHAROS, Light Conversion, Vilnius, Lithuania) with a 0.2 ps pulse duration.

The differential transmission decays (pump–probe, PP) were measured using pulsed 527 nm excitation (10 ps, Nd:YLF laser, second harmonic) and a 1550 nm CW probe (provided by a single-mode laser from Eblana Photonics (Dublin, Ireland), 50 mW). The temporal transmission of the sample after excitation was detected using a 2 GHz detector with a low-noise amplifier and was recorded by a 6 GHz oscilloscope (LeCroy SDA6000, Heidelberg, Germany). The setup details can be found in [18].

The absorption spectra of the studied sample were measured using a Perkin Elmer Lambda 950 UV-NIR absorption spectrometer (Waltham, MA, USA); details about the experimental arrangement were reported elsewhere [8]. The sample photoconductivity spectra and decay times were taken from Ref. [8] for comparison. Therein, the same second-harmonic Nd:YLF laser excitation sources were used, with the detection system providing a 100 ps temporal resolution.

## 3. Results

### 3.1. Absorption and Photoluminescence Spectra

**Figure 1:**
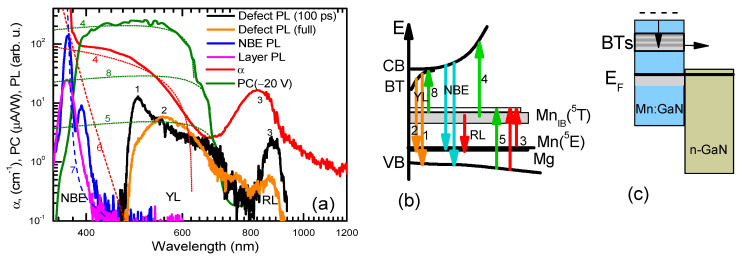
(**a**) The PL spectra of SI Am-GaN:Mn with conductive epitaxial GaN epilayer. For comparison purposes, the absorption and photoconductivity spectra adapted from Ref. [8] are also shown. Near-band emission (NBE) PL was excited by 330 nm, while defect PL was excited by 400 nm wavelength at 300 K. Note: 1—fast band tail-to-hole transitions; 2—band tail-to-Mg^0^ transitions; 3—internal Mn-IB-to-VB transitions; 4—Mn-IB-to-CB transition; 5—VB-to-free Mn-IB-state transition; 8—Mn-IB-to-band tail transition; 6—Urbach tail (95 meV); 7—high purity GaN band-edge absorption. (**b**) Tentative optical transitions via impurity states of Mn and Mg atoms. Note: Mg—acceptor; YL—yellow luminescence; RL—red luminescence. (**c**) Scheme of the electron transitions in the SI-n junction.

The PL spectra were measured, and the results are shown in Figure 1. We compared the measurements with the absorption and photoconductivity spectra reported in Ref. [8], analyzing a wide absorption band in the spectrum range of 400–700 nm with a peak at 850 nm [8]. Most processes ascribed to the Mn defect transitions are illustrated schematically in Figure 1b. The 850 nm peak corresponds to the internal spin-allowed electron transitions in Mn ions ^5^E → ^5^T, with a 1.5 eV energy [19]—process 3. The wide band is explained by the transitions from VB to the empty Mn intermediate band states (Mn-IB)—process 5, transitions from Mn-IB to CB—process 4, transitions from Mn-IB to band tails (BTs) induced by shallow donors (O, Si, C (on Ga site), and H) and a deep Mn interstitial donor at 1.0 eV below CB [20]—process 8. Process 6 describes the transitions from VB to BTs and shallow donors. The photoconductivity band has a maximum in the 420–550 nm range. Near the bandgap of GaN, the photoconductivity drops as carriers are generated only near the surface and cannot pass through the semi-insulating Am-GaN sample. The photoluminescence spectra correlate with the absorption and photoconductivity spectra. The absorption peak red edges coincide with the PL spectra red edges where the radiative transitions occur (red luminescence (RL) and yellow luminescence (YL)). The PL spectra quickly change after photo-excitation due to the capture of free electrons and holes to the BTs and acceptors, respectively. The near-band-edge emission (NBE) has an additional 3.16 eV sub-bandgap peak NBE2 in the SI GaN:Mn, related to some shallow impurity and defect states [21,22,23], from a large amount of which one can be attributed to Mg^0^ acceptors [21]. Its peak corresponds to 214 meV lower energy with respect to the band-to-band transition peak (NBE1). The Mg and C are acceptors with activation energies of 223 meV [24] and 880 meV (on the N site) [25,26], respectively.

An equation: α = α_0_exp((hν – E_g_)/E_U_), with E_U_ = 95 meV (here, E_g_ = 3.4 eV is the GaN bandgap), fits the Urbach edge in the absorption spectra (Figure 1a). The E_U_ value was determined from the YL thermal activation (see below). Similar ~100 meV Urbach energy was found in [27] for Mn-doped GaN. The absorption coefficient was fitted with the equation α = coef × (hν – E_te_)^1/2^/hν (process 4), while photoconductivity was with α = coef × (hν – E_te_)^1/2^/(hν)^2^(4, 5, 8 processes) with threshold values of E_te_ = 2.0 ± 0.05 eV, 1.75 ± 0.05 eV, and 1.82 eV for the 4, 5, and 8 processes, respectively [8]. Process 4 is by an order of magnitude stronger than process 8, which is stronger than the photoconductivity of electrons in the band tails. The electron transfer in the sample is depicted in Figure 1c. Data for the energy diagram were calculated using the Fermi level positions [8,28].

### 3.2. Photoluminescence Decay Characterization

**Figure 2:**
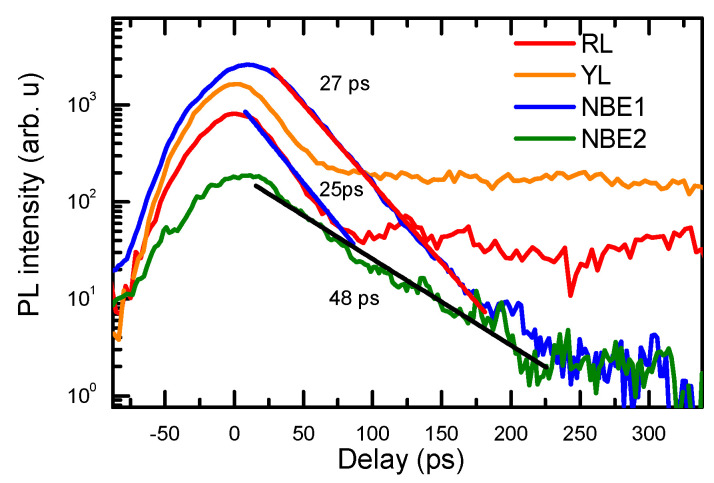
Fast BB (NBE1), e^−^Mg^0^(NBE2), YL, and RL TRPL decay at 300 K.

The initial PL decays of the observed bands are provided in Figure 2. The decay time of the near-band emission (NBE) in Am-GAN is very fast (band-to-band (BB) NBE1), the peak decay time is 27 ps, and the e^−^ to Mg^0^ transition decay time is larger—39 ps (NBE2; see Figure 2). A similar electron decay time of 28 ps was observed in the Mn-doped GaN by femtosecond pump–probe measurements [15]. Holes from the valence band for the BB transitions are captured to Mg^−^ states, and the BB transitions disappear (see Figure 1b inset). Electron transitions to Mg^0^ states dominantly quench due to electron capture to the nonradiative traps and thermalization to BTs.

**Figure 3:**
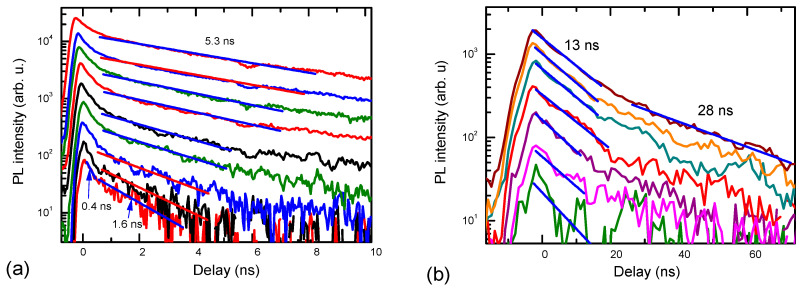
Slow YL (**a**) and RL (**b**) decays at 300 K. Straight lines show exponential fits. Excitation intensities are the same as in Figure 4.

**Figure 4:**
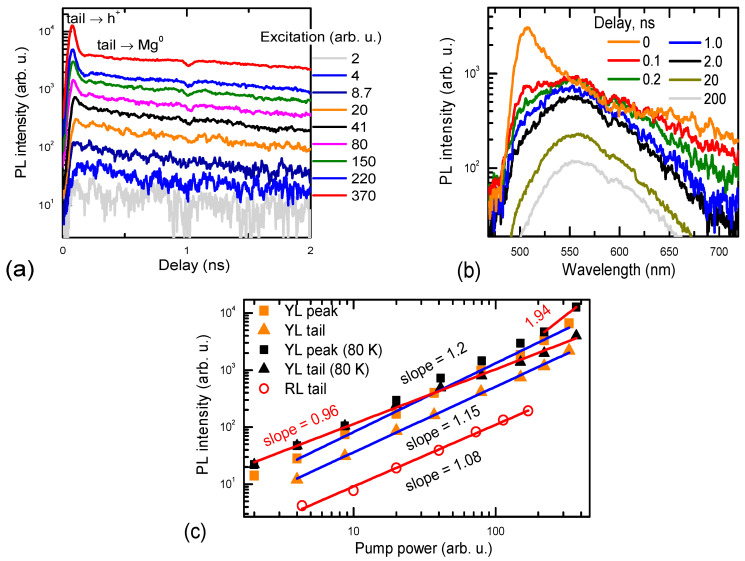
YL excitation-dependent decay (**a**), and time-dependent PL spectra at 80 K (**b**), YL, and RL band intensity vs. excitation (**c**).

The red and yellow luminescence fast decay parts (Figure 2) are related to the electrons in the BTs and electrons in the Mn-IB recombination with free holes. The initial fast YL decay part shows band tail-to-free hole transitions (peak 1), and at longer delays (Figure 3), holes are captured to Mg^−^, where band tail-to-Mg^0^ transitions become dominant (peak 2). Fast peak 1 is more pronounced at higher excitations (Figure 1a and Figure 4b) when the electron lifetimes increase, and the capture rate to Mg^−^ acceptors is reduced due to their plausible saturation. The YL band peak quickly shifts to the red spectrum side by ~250 meV, which corresponds to the Mg activation energy. Indeed, these band tail-to-Mg^0^ transitions decay much slower (see Figure 3) and reflect electron recombination in the BTs. The YL decays at low excitations (Figure 3a) show a fast initial part of 0.4 ns, and furthermore, the decay time slows down to 1.6 ns. During the fast initial part, the spectrum exhibits a drop in intensity on the red energy edge (see Figure 4b), which can be attributed to the decay of BT-to-Mn-IB transitions, as empty Mn-IB states disappear due to the filling of them with electrons thermalizing to these states (i.e., the fast decay (0.4 ns) is related to the filling time of empty Mn-IB states after excitation). Similar, fast 0.27 ns and slow 1.68 ns electron decay times were observed by femtosecond pump–probe measurements [15] and are attributed to the 3 eV → 1.9 eV electron transitions in the band tails and electron decay time in Mn-IB, respectively. The YL superlinear growth with excitation can be explained by the saturation of the nonradiative traps with holes (electrons). Peak 3, showing electrons from Mn-IB-to-free hole transitions, also decays quickly (see Figure 4a). Its decay time weakly depends on the excitation. Still, some of the peak 3 signal is observed at a slow delay, which can be related to the Mn-IB transitions to thermally activated holes from Mg^0^ acceptors. The latter occurs with an ~12–20 ns time (Figure 3b).

With the excitation increase, the YL decay time becomes slower, up to 5.3 ns (Figure 3a), which can be explained by the saturation with electrons of the nonradiative defects, which are typically V_Ga_-complexed with oxygen and hydrogen [21], and a plausible saturation of free Mn-IB states or C acceptors. In this saturated regime, electron and hole decay can be limited by their capture to the nonradiative defects. By thermal ionization, the acceptor provides holes to the valence band; those holes are captured to the nonradiative defects filled with electrons and can recombine nonradiatively (see Section 4 for more details).

Figure 4 shows the excitation-dependent YL decay. In Figure 4a, a fast part is emerging at high excitations due to the band tail-to-free hole transitions when the Mg acceptors are becoming saturated. Simultaneously, the PL spectrum blue-shifts by acceptor energy at short delays (Figure 4b). At low T = 80 K, charged Mg^−^ states capture holes faster than the nonradiative defects; thus, the slow decay amplitude slope is closer to unity (Figure 4c). A fast YL peak at 80 K also shows a superlinear slope of 1.94 at the highest excitation due to efficient bimolecular electron–hole recombination [29] (a two-step absorption generates both electrons and holes; at low temperatures, the direct bimolecular recombination is more efficient due to the rate coefficient dependence on temperature as T^−3/2^ [30]). The RL at low temperatures was not measurable due to plausibly smaller hole activation and a much faster capture to Mg^−^.

**Figure 5:**
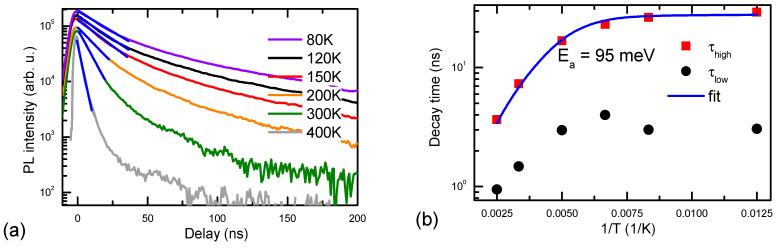
YL decays at high excitation at different temperatures (**a**). Initial YL lifetime vs. T at high and low excitations (**b**); Straight lines in (**a**) show exponential fits, while in (**b**) provides activation fit for BTs, function τ = 1/(a + b × exp(−E_a_/kT)) was applied for fitting; a = 3.6 × 10^7^ s^−1^, b = 4 × 10^9^ s^−1^.

The temperature-dependent YL decays are shown in Figure 5. They become considerably slower at low temperatures, reaching 30 ns. The observed activation energy of 95 meV can be ascribed to electron activation in the BTs, as at RT, the YL lifetime drops to 3.5 ns, which is much lower than the hole decay time observed in the RL process (~20 ns, Figure 3b). The initial amplitude is not changed significantly vs. temperature due to the similar number of excited carriers (shown at a maximum excitation intensity) and the indirect phonon-assisted nature of the BTs-to-Mg^0^ transition process.

### 3.3. Pump Probe Decay Characterization

**Figure 6:**
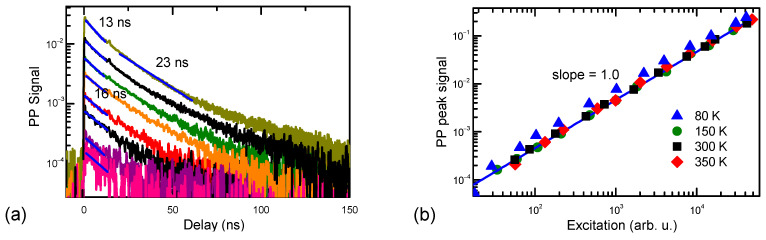
PP decays vs. pump intensity at 527 nm and 1550 nm probes at 300 K (**a**); PP signal excitation dependence linearity (**b**).

**Figure 7:**
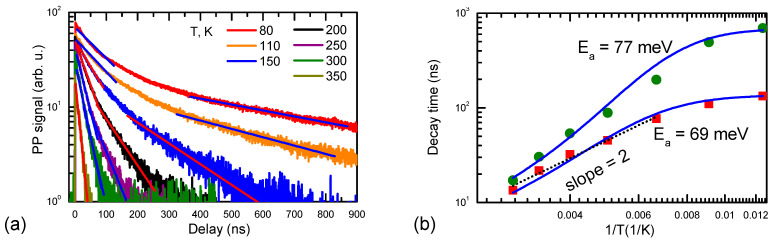
Temperature-dependent PP decays (**a**) and their initial decay time thermal activation (**b**). Straight lines in (**a**) show exponential fits, while in (**b**) function τ = 1/(a + b × exp(–E_a_/kT)) was applied for fitting; a = 7.5 × 10^6^ s^−1^, b = 7 × 10^8^ s^−1^, with Ea = 69 meV and 77 meV for the fast and the slow parts, respectively. Initial (red points) and slow (green points) decay parts provide almost the same activation energy.

The PP decay was investigated by varying pump intensities of 527 nm and using a 1550 nm probe beam. The results are shown in Figure 6. The initial decay part is faster, up to 13 ns, which indicates slow defect filling or inhomogeneity of their distribution. This decay time is related to the hole’s lifetime, as holes have a much larger 1550 nm photon absorption cross-section than the free electrons with σ_e_~10^−18^ cm^2^ [31]. The hole absorption cross-section is by order of magnitude larger, σ_h_~3 × 10^−17^ cm^2^ [32]. On the other hand, the pump–probe decay time was 22 ns (Figure 6a). This was plausibly related to the hole relaxation in Mg states. In Mg-doped GaN Mg^0^ states (generated after hole capture to Mg^−^), the photo-ionization cross-section at 1550 nm can be evaluated as ~5 × 10^−17^ cm^2^ [31]. For the experimentally observed PP decays, we calculated an 8 × 10^−19^ cm^2^ absorption cross-section (the generated carrier (electron) density was estimated as ΔN = αI_exc_/hν, and I_exc_ is the excitation intensity). Such a weak response can be explained by the relatively weak generation of holes during the photo-excitation at 532 nm (absorption process 5 is approximately 50 times weaker than process 4; see Figure 1a,b). In support of this hypothesis, the RL has a similar and weakly excitation-dependent lifetime. The RL slow decay part shows the Mn-IB transition to the Mg^0^ acceptor (the latter decay is also monitored by PP). The PP decay time strongly reduces with temperature (the activation energy is 69 meV; Figure 7) as the Mg^0^ acceptors thermally ionize and provide holes for recombination on the nonradiative traps. The PP decay amplitude is linear on the excitation and shows an almost constant vs. excitation lifetime at RT (Figure 6). Amplitude linearity indicates that the generated hole density is proportional to the excitation intensity.

## 4. Discussion

The sub-bandgap 532 nm exciting photons transfer electrons from the Mn-IB to the BTs, from VB to Mn-IB, and from Mn-IB to CB (the electrons quickly thermalize from CB to BTs). In this case, the sample resistivity temporarily drops by 10 orders of magnitude, demonstrating very small electron mobility, whose value was found in the range of 2.0 cm^2^/Vs, resulting in a weak photo-responsivity of GaN material [8].

Both RL and PP are determined by holes, which are captured by Mg^−^ and C^−^ acceptors and thermally activate slowly, leading to delayed decay. The capture cross-section for C^-^ acceptors 9 × 10^−7^ cm^3^/s [33] at 10^17^ cm^−3^-doping provides a 10 ps decay time, which can explain the fast hole disappearance in the Am-GaN sample. The ionized Mg^−^ acceptors already have a high capture cross-section for the holes; thus, hole disappearance is determined by the concurrent hole capture by Mg^−^ and C^−^ acceptors. The carbon defect has a rather constant vs. T capture cross-section [34]; therefore, the capture of holes to the Coulombic Mg^−^ acceptors is more efficient at low temperatures, as their capture cross-section should increase as T^−2^ (see the slope in Figure 7b). This effect can explain the observed decay time activation with temperature; Mg thermal activation should start at a higher T due to a larger activation energy value. The linear YL slope at all temperatures was observed due to ineffective hole generation during excitation (only 2% of the absorbed photons, up to ~10^16^ cm^−3^), which is much lower than the acceptor density (Figure 4c). Indeed, even a smaller fraction of Mg was activated to provide holes.

**Figure 8:**
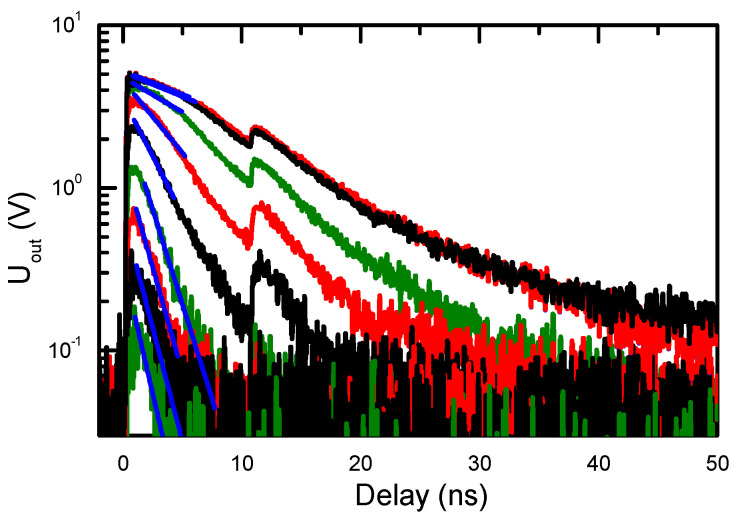
Photoconductive signal decays at different laser excitation pulse energies (corresponding to the carrier densities shown in Figure 9) at 16 V bias voltage at 300 K. The second decay peak at the 10 ns time mark corresponds to the decay excited by a parasitic (by a magnitude weaker) laser pulse arriving after the main pulse; solid lines are exponential fits providing PC lifetime in Figure 9.

**Figure 9:**
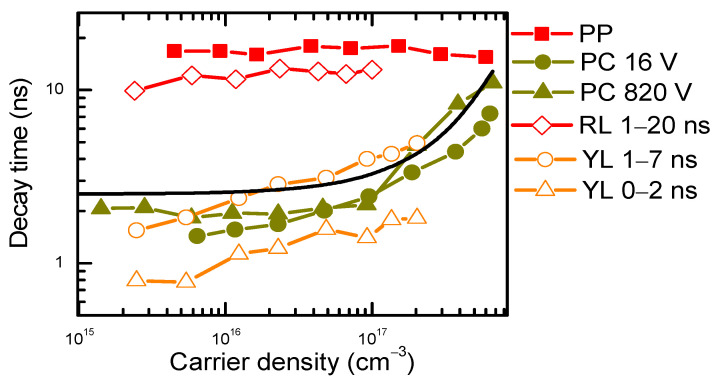
Decay time vs. excited carrier density by time-resolved pump–probe (PP), photoluminescence (PL), and photoconductivity (PC) [8]. For RL and YL, different time windows are indicated where lifetime was determined in Figure 3.

The photoconductivity superlinear vs. excitation slope 1.1–1.3 (observed in Ref. [8]) is very close to the YL slope of 1.15 at 300 K (see Figure 4c). At low temperatures, the YL slope for the slow tail is reduced to unity as nonradiative traps presumably start capturing holes slower than Mg^−^ acceptors (the Coulombic trap Mg^−^ capture cross-section strongly increases at lower temperatures). Therefore, the superlinear photoconductivity slope can be ascribed to the impact of traps at low excitations. The PC decays were measured and shown in Figure 8. It used to determine the PC lifetime, the results of which are shown in Figure 9. The excitation-dependent decay times confirm YL’s relation to the electron photoconductivity, both having similar decay times that increase with excitation due to the defect saturation. The PC lifetime and YL lifetime (determined from the PL decays in Figure 3) have similar excitation dependences, relating them to the electron in BT decay. Saturation of the C traps can explain the decay time increase with excitation. The PP and RL lifetimes coincide quite well, and both have weak excitation dependence; therefore, they can be explained by the hole population decay due to the thermal activation from Mg or C acceptors.

At different bias voltages, the photoconductivity decay time increase with excitation was very similar [8], indicating it is limited by the internal properties of Am-GaN. Photoconductivity and the YL decay time’s (τ_dec_) increase with excitation can be explained by the saturation of carbon defects C_N_ [35,36] with a density of approximately N_t_~N(C_N_) = 4 × 10^17^ cm^−3^ in the investigated sample. For the 0/+ state, the C_N_ defect has a capture coefficient of C_n2_ = 10^−9^ cm^3^/s for electrons and C_p2_ = 10^−10^ cm^3^/s for holes [35]. Then, the capture time for the electrons is τ_e_ = 1/(C_n2_N_t_) = 2.5 ns, while for holes, it would be much larger, τ_h_ = 1/(C_p2_N_t_) = 25 ns. A similar value of the hole’s lifetime is observed for the RL and PP. The experimental electron PC decay time can be described by the following equation: 1/τ_dec_ (ΔN) = (1 – exp(−ΔP/N_t_))/τ_h_ + exp(−ΔN/N_t_)/τ_e_ [37], where the hole density is ΔP = ΔN/50. The fit for this equation is shown by the solid line in Figure 9. At a low excitation, τ_dec_~τ_e,_ the electrons fill empty C_N_ defects, while at high excitations, τ_dec_~τ_h,_ the recombination is limited by the hole’s capture to defects, which are filled. The C_N_ capture cross-sections have huge variations in the literature, providing high imprecisions. Therefore, in the saturated regime, the electron and hole decay can also be limited by their capture to other nonradiative GaN defects, based on oxygen and hydrogen V_Ga_ complexes [38]. There are many other different defects in the sample; thus, they can have some impact on the obtained results.

Therefore, gaining control of the C and Mg impurity densities may be essential for improving the Am-GaN operation by enhancing the electron lifetime and hole mobility, respectively. Moreover, the ultrashort intrinsic (~10 ns) hole lifetime is highly desirable for the fast reverse-recovery performance of vertical GaN power rectifiers [39].

## 5. Conclusions

Using photoluminescence and pump–probe spectroscopy, the generated hole and electron lifetimes were evaluated to be 10–20 ns and 1–10 ns, respectively, in the Am-GaN:Mn semiconductor. The electrons and holes, photo-generated in the conduction and valence bands, quickly thermalized to the conduction band tails and Mg acceptors, respectively. The electron lifetime in the band tails increased with an excitation intensity from 1.7 ns to 11 ns due to the saturation of carbon defects, while the hole lifetime remained constant. The YL and PP trace decay times showed thermal activation energies of 95 meV and 65 meV, respectively. They dominantly indicated the activation of electrons in the band tails and holes from Mg^0^ acceptors, respectively. The observed fast restoration of the electron and hole density (<20 ns) is very attractive for applications in high-voltage and high-frequency vertical GaN power devices.

## Data Availability

The original contributions presented in this study are included in the article. Further inquiries can be directed to the corresponding authors.
